# The Anxious Bipolar Phenotype: Clinical Complexity and Treatment Resistance

**DOI:** 10.21203/rs.3.rs-8503080/v1

**Published:** 2026-01-12

**Authors:** Balwinder Singh, Ada Man-Choi Ho, Brandon Coombes, Francisco Romo-Nava, Alfredo Cuellar-Barboza, Manuel Gardea-Reséndez, David Bond, Miguel Prieto, Marin Veldic, Richard Pendegraft, Susan McElroy, Joanna Biernacka, Mark Frye

**Affiliations:** Mayo Clinic; Mayo Clinic; Mayo Clinic; Lindner Center of HOPE; Mayo Clinic; Mayo Clinic; Johns Hopkins University; Universidad de Los Andes, Chile; Mayo Clinic; Mayo Clinic; Lindner Center of HOPE; Mayo Clinic; Mayo Clinic

**Keywords:** Bipolar disorder, anxiety disorders, comorbidity, pharmacotherapy, treatment response, mood stabilizers, antidepressants

## Abstract

**Background:**

Anxiety disorders (ANX) affect 30–60% of individuals with bipolar disorder (BD), yet limited research has systematically examined clinical characteristics and treatment patterns in this comorbid population. This study investigated demographic, clinical, and pharmacotherapeutic differences between individuals with BD with and without comorbid ANX.

**Methods:**

Cross-sectional data from 2,225 adults with BD enrolled in the Mayo Clinic Bipolar Disorder Biobank were analyzed. Participants were assessed for comorbid ANX, demographics, clinical characteristics, medication use, and treatment response using the Alda-A scale.

**Results:**

Overall, 61% (n = 1,366) had comorbid ANX. Individuals with BD + ANX were younger (40.4 vs. 43.6 years, p < 0.001), more likely female (66.6% vs. 54.8%, p < 0.001), and exhibited higher rates of rapid cycling (64.2% vs. 45.2%, p < 0.001), suicide attempts (40.4% vs. 24.8%, p < 0.001), substance use disorders (63.5% vs. 54.8%, p < 0.001), and somatic comorbidities (MCIRS: 6.68 vs. 5.42, p < 0.001). Pharmacotherapeutically, BD + ANX individuals were less likely to receive lithium (37.1% vs. 47.8%, p = 0.005) and valproic acid (21.7% vs. 29.6%, p = 0.047), but more likely to receive antidepressants (53.8% vs. 39.5%, p < 0.001), benzodiazepines (39.9% vs. 26.6%, p < 0.001), and gabapentinoids (8.5% vs. 4.5%, p < 0.001). Notably, 17.3% of BD + ANX individuals received antidepressants without mood stabilizer coverage. Treatment response (Alda-A) scores were significantly lower in BD + ANX for lithium (4.91 vs. 6.05, p < 0.001), mood-stabilizing anticonvulsants (5.09 vs. 6.22, p < 0.001), and second-generation antipsychotics (4.67 vs. 5.73, p < 0.001). Similar patterns were observed in both BD-I and BD-II subtypes.

**Conclusions:**

Individuals with BD + ANX represent a more severely affected subgroup with distinct prescribing patterns favoring antidepressants over mood stabilizers and attenuated mood stabilizers response. These findings highlight the need for anxiety-informed treatment algorithms recognizing anxiety comorbidity as a negative prognostic factor.

## INTRODUCTION

A significant challenge in effectively managing bipolar disorder (BD) is the high prevalence of comorbidities, particularly anxiety disorders (ANX) (Pavlova et al., 2015; Singh et al., 2024), with clinical estimates up to 60% (Kinrys et al., 2019; McIntyre et al., 2006; Pavlova et al., 2015; Singh et al., 2025b). Those with ANX are at increased risk for developing substance use disorders (SUD) and frequently self-medicate with alcohol (Smith and Book, 2008). Previous research has indicated that BD with comorbid ANX (BD + ANX) correlates with substantial functional impairment, risk for suicidality, and poor quality of life (Burdick et al., 2022; Simon et al., 2004). A recent systematic review reported the current prevalence of ANX in BD at approximately 38%, with generalized anxiety disorder (GAD) being the most common subtype (Yapici Eser et al., 2018). Although some studies suggest the lifetime risk of ANX may be as high as 60–85% (Merikangas et al., 2007; Mitchell et al., 2013), there remains a lack of consistent data on prevalence rates across bipolar I and bipolar II subtypes, as well as on associated clinical outcomes (Cullen et al., 2021; Galimberti et al., 2020; Kauer-Sant’Anna et al., 2009; Keck et al., 2006; Kinrys et al., 2019; Schaffer et al., 2012; Vazquez et al., 2014). There is a paucity of recent data examining pharmacotherapeutic approaches and treatment response differences among bipolar individuals with (BD + ANX) and without lifetime anxiety (BD + NoANX).

A critical discussion within the field of BD concerns the prescription rates of monoaminergic antidepressants (Elmosalamy et al., 2025; Park et al., 2022), which may vary between 30–70%, depending on the region and study populations (Singh et al., 2024; Yocum and Singh, 2025). Evidence suggests that individuals with ANX are frequently prescribed monoaminergic antidepressants (Yatham et al., 2018), which may increase the risk of affective switching in BD. This raises the question of whether pharmacotherapy patterns differ between individuals with BD + ANX and BD + NoANX, with the former typically receiving higher rates of antidepressant prescriptions (Keck et al., 2006). Understanding these prescribing trends can inform clinical practices and contribute to strategies aimed at enhancing treatment outcomes in future research.

Recent data from the Global Bipolar Cohort Collaboration showed prevalence rates of comorbid ANX as high as 60% in the North American cohorts compared to 30% in the European cohorts (Singh et al., 2024). We also observed regional variations in prescription patterns; however, data regarding the BD + ANX phenotype were not available. Leveraging data from the Mayo Clinic Bipolar Disorder Bank, this study examined the differences in clinical and demographic characteristics as well as pharmacotherapeutic prescription patterns among individuals with BD + ANX and BD + NoANX. Furthermore, we analyzed prescription patterns among individuals with bipolar I (BD-I) and bipolar II disorder (BD-II) with and without ANX, as those with BD-II tend to be prescribed more monoaminergic antidepressants (Singh et al., 2024).

## METHODS

Detailed information about the Mayo Clinic Bipolar Disorder Biobank has been previously published (Frye et al., 2015). In summary, cross-sectional data was collected from study participants at enrollment (Gardea-Resendez et al., 2022; Pahwa et al., 2021b). Participants were recruited from five sites: Mayo Clinic, Rochester, Minnesota; Lindner Center of HOPE/University of Cincinnati College of Medicine, Cincinnati, Ohio; University of Minnesota, Minneapolis, Minnesota; Universidad Autónoma de Nuevo León, Mexico; and Universidad de los Andes, Chile. The inclusion criteria consisted of adults aged 18–80 with BD who spoke English at the U.S. sites and Spanish at Mexico and Chile sites, provided informed consent, and met DSM-IV-TR criteria for BD-I/BD-II or schizoaffective BD. Participants exhibiting active psychosis or suicidal ideation were excluded.

At the time of study enrollment, data were collected on demographics, family history, psychiatric conditions (including adult and childhood attention deficit hyperactivity disorder [ADHD], anorexia nervosa, bulimia nervosa, binge eating disorder (BED), GAD, obsessive-compulsive disorder (OCD), panic disorder, post-traumatic stress disorder (PTSD), and social anxiety disorder (SAD), medications, and somatic comorbidities. For this study, GAD, panic disorder, SAD, and phobias were categorized under the ANX category. Since most ANX persist as psychiatric diagnoses throughout an individual’s life, our study concentrated on lifetime ANX diagnoses. The overall burden of medical illness was measured using the Modified Cumulative Illness Rating Scale (MCIRS) (Salvi et al., 2008), with psychiatric comorbidity data excluded. Data for additional somatic comorbidities were extracted using structured surveys.

We collected data on current and lifetime prescriptions for second-generation antipsychotics (SGAs) and mood stabilizers, including lithium and mood-stabilizing anticonvulsants (MSACs) such as valproate, carbamazepine, and lamotrigine. Additional data were obtained for first-generation antipsychotics (FGAs), monoaminergic antidepressants—including selective serotonin reuptake inhibitors (SSRIs), serotonin-norepinephrine reuptake inhibitors (SNRIs), and tricyclic antidepressants (TCAs)—as well as benzodiazepines, gabapentinoids (gabapentin or pregabalin), stimulants, wakefulness-promoting agents, and dopamine agonists. We examined patterns of polypharmacotherapy, defined as the concurrent use of two or more SGAs or mood stabilizers, and assessed the frequency of monoaminergic antidepressant prescriptions without a concurrent mood stabilizer.

Treatment response to lithium, SGAs, and MSACs was assessed using the Alda-A score from the Alda Scale (Grof et al., 2002). The Alda-A score measures clinical improvement in illness severity during the treatment, rated from 0 (no improvement or worsening) to 10 (full recovery or absence of symptoms). Although initially developed to retrospectively measure response to lithium, it has been modified to assess response to other mood stabilizers in previous studies (Cuellar-Barboza et al., 2020; Ho et al., 2020; Joseph et al., 2023; Pahwa et al., 2021a; Singh et al., 2025a).

### Statistical analysis

Clinical and demographic characteristics were compared between participants with and without a lifetime anxiety diagnosis (BD + ANX and BD + NoANX, respectively) using the *arsenal* package in R. The *modelsum* function was applied to perform multiple univariate tests, using linear models for continuous variables and logistic models for categorical variables. All models were adjusted for age, sex, and recruitment site (Mayo Clinic, Lindner Center of HOPE, University of Minnesota, Chile, or Mexico). Given the large number of comparisons, a threshold of *p* < 0.001 was used to define statistical significance. Analyses were conducted using R version 4.2.2.

## RESULTS

The study cohort consisted of 2,225 adults with BD (1,451 BD-I, 723 BD-II, 51 schizoaffective disorder BD). The mean age was 41.6 years; 62.1% were female, 84.0% White, and 12.4% Hispanic (Table-1). Rapid cycling was prevalent in 57.1% of individuals, with a history of psychosis in 39.6%, and 38.1% had early onset of BD.

**Figure 1:**
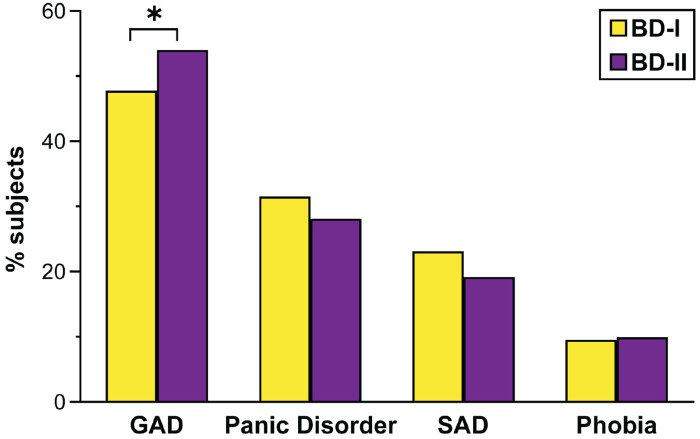
Prevalence of Anxiety Disorders, PTSD, and OCD in Bipolar I and Bipolar II disorders. GAD: generalized anxiety disorder; OCD: obsessive-compulsive disorder; PTSD: post-traumatic stress disorder; SAD: social anxiety disorder. *p < 0.05.

Ninety percent of ANX data came from the US, with the rest from Mexico and Chile. Overall, 61.4% (n = 1366) had ANX, with similar rates in BD-I (66.7%) and BD-II (64.3%). Among individuals with BD-I and BD-II, the most common ANX was GAD (50%), followed by panic disorder (30.3%), SAD (21.8%), and phobias (9.6%). There is a trend toward a higher prevalence of GAD in BD-II compared to BD-I (54.0% vs. 47.7%, *p* = 0.021), while rates of other ANX disorders were similar across BD subtypes (Figure-1).

### Comparison of individuals with BD + ANX (n = 1366) and BD + NoANX (n = 859)

Table 1 shows the comparisons of demographic and clinical characteristics between individuals with BD + ANX and BD + NoANX, adjusted for age, sex, and recruitment site. Individuals with BD + ANX were younger (mean age 40.4 vs. 43.6 years, *p* < 0.001) and more likely female (66.6% vs. 54.8%, p < 0.001) compared to those with BD + NoANX. They exhibited higher rates of rapid cycling (64.2% vs. 45.2%, *p* < 0.001), BED (12.3% vs. 7.5%, *p* < 0.001), PTSD (34.6% vs. 12.6%, p < 0.001), OCD (21.3% vs. 8.5%, *p* < 0.001), and SUDs (63.5% vs. 54.8%, *p* < 0.001). Suicidal attempts (40.4% vs. 24.8%, *p* < 0.001), tobacco use disorder (43.1% vs. 35.1%, *p* < 0.001), cocaine use disorder (16.6% vs. 10.6%, *p* < 0.001), alcohol use disorder (42.3% vs. 36.0%, *p* = 0.002), cannabis use disorder (33.3% vs. 24.4%, *p* < 0.001), methamphetamine use disorder (10.4% vs. 7.0%, *p* = 0.002), and opioid use disorder (12.8% vs. 6.9%, *p* < 0.001) were more prevalent in individuals with BD + ANX. A family history of BD (51.5% vs. 40.3%, *p* < 0.001), anxiety (68.5% vs. 49.7%, *p* < 0.001), depression (84.8% vs. 72.6%, *p* < 0.001), and alcohol use disorder (54.0% vs. 40.8%, *p* < 0.001) was also more common. Individuals with BD + ANX had higher MCIRS scores (6.68 vs. 5.42, *p* < 0.001), more migraines (36.8% vs. 18.9%, *p* < 0.001), and more current (2.86 vs. 2.50, *p* < 0.001) and lifetime use of psychotropics (7.27 vs. 5.74, *p* < 0.001).

### Pharmacotherapeutic differences among individuals with BD + ANX (n = 1366) and BD + NoANX (n = 859)

**Figure 2:**
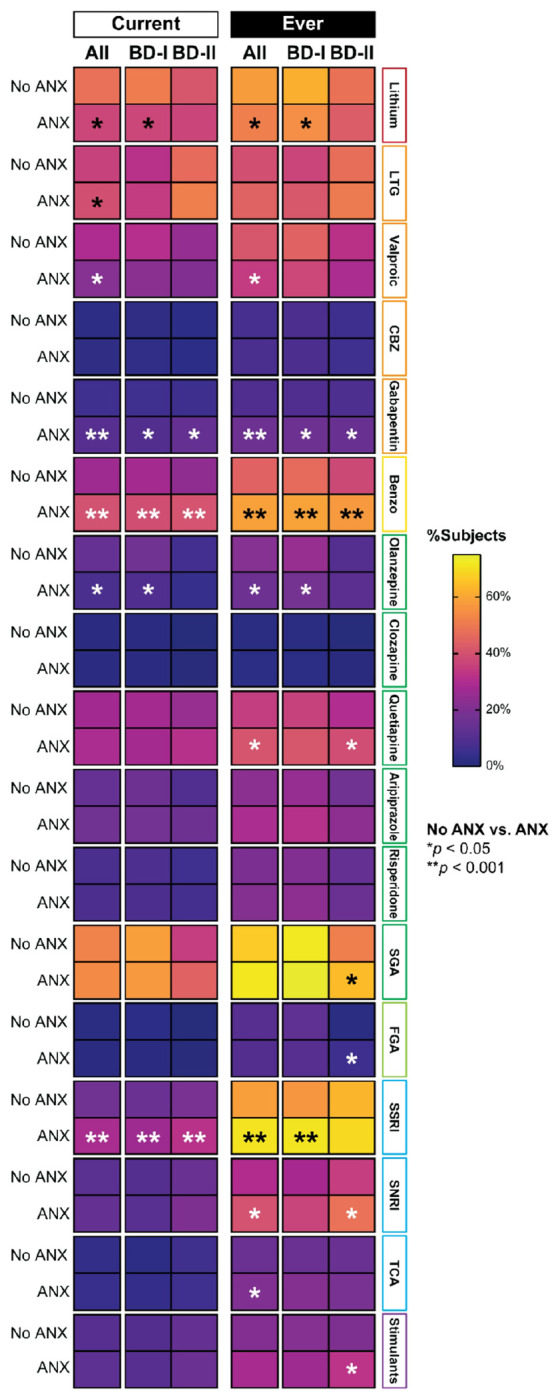
Current and lifetime pharmacotherapeutic prescribing patterns among individuals with bipolar disorder, with and without comorbid anxiety (ANX), by Bipolar I and Bipolar II subtypes. Benzo: benzodiazepines; CBZ: carbamazepine; FGA: first-generation antipsychotics; SGA: second-generation antipsychotics; SNRI: serotonin-norepinephrine reuptake inhibitor; SSRI: selective serotonin reuptake inhibitor; TCA: tricyclic antidepressants.

Individuals with BD + ANX (*n* = 1366) differed significantly from those with BD + NoANX (*n* = 859) across multiple pharmacotherapeutic variables (Table 2, Fig. 2). Individuals with BD + ANX were less likely to be currently prescribed lithium (37.1% vs. 47.8%, *p* = 0.005) and showed a trend towards lower valproic acid use (21.7% vs. 29.6%, *p* = 0.047), but more likely to receive gabapentinoids (8.5% vs. 4.5%, *p* < 0.001) and benzodiazepines (39.9% vs. 26.6%, *p* < 0.001). They were also significantly more likely to receive antidepressant treatment (53.8% vs. 39.5%, p < 0.001), including two or more concurrent antidepressants (13.0% vs. 6.9%, p < 0.001), particularly SSRIs (28.8% vs. 16.8%, p < 0.001), and to be prescribed antidepressants without a concomitant mood stabilizer (17.3% vs. 9.7%, p < 0.001). Olanzapine prescriptions were lower among BD + ANX individuals (8.0% vs. 14.0%, *p* = 0.008), while there was a trend toward slightly higher lamotrigine use (39.7% vs. 35.8%, *p* = 0.027). No significant differences were observed for carbamazepine, quetiapine, FGAs, thyroid hormones, stimulants, or dopamine agonists.

Lifetime treatment patterns mirrored these trends: individuals with BD + ANX show a trend toward lower use of lithium (51.3% vs. 57.9%, *p* = 0.030) and valproate (34.6% vs. 41.3%, *p* = 0.013) but were more likely to have received gabapentinoids (15.6% vs. 8.8%, *p* < 0.001), benzodiazepines (58.8% vs. 44.4%, *p* < 0.001), SSRIs (70.6% vs. 58.5%, *p* < 0.001), and SNRIs (40.1% vs. 30.5%, p = 0.002). They were also more likely to have been treated with antidepressants without a mood stabilizer (14.6% vs. 8.7%, *p* < 0.001).

### Pharmacotherapeutic differences among individuals with BD-I and ANX (n = 878) and those without ANX (n = 573)

Among individuals with BD-I (ANX *n* = 878; No ANX *n* = 573), significant differences were observed in several pharmacotherapeutic variables (**Supplementary Table 1**, Fig. 2). Individuals with BD-I + ANX were less likely to be currently prescribed lithium (37.1% vs. 50.7%, *p* = 0.009), while valproic acid (22.4% vs. 31.2%, *p* = 0.137) and lamotrigine use (34.6% vs. 31.8%, *p* = 0.092) showed no statistically significant differences. There was a trend toward higher use of gabapentinoids (7.8% vs. 4.8%, *p*= 0.041) and significantly higher use of benzodiazepines (39.4% vs. 27.7%, *p* < 0.001). Olanzapine prescriptions trended lower among ANX individuals (9.4% vs. 16.9%, *p* = 0.023), while no significant differences were observed for carbamazepine, quetiapine, aripiprazole, risperidone, FGAs, or SGA overall. Regarding antidepressants, individuals with BD-I + ANX were more likely to be prescribed any antidepressant (48.8% vs. 37.0%, p < 0.001), two or more concurrent antidepressants (10.5% vs. 4.0%, p < 0.001), and SSRIs (26.5% vs. 15.5%, p < 0.001), and to receive antidepressants without a concomitant mood stabilizer (15.9% vs. 7.7%, p = 0.001). Thyroid hormone use was also lower among ANX individuals (17.2% vs. 27.2%, *p* = 0.014).

Lifetime treatment patterns reflected similar trends: individuals with BD-I + ANX show a trend toward lower lithium use (54.6% vs. 61.9%, *p* = 0.035) and a trend toward higher use of gabapentinoids (15.6% vs. 9.0%, *p* = 0.015), benzodiazepines (59.2% vs. 46.7%, *p* < 0.001), SSRIs (70.9% vs. 56.6%, *p* < 0.001), and quetiapine (41.3% vs. 35.7%, *p* = 0.052), with only benzodiazepines and SSRIs reaching statistical significance.

### Pharmacotherapeutic differences among individuals with BD-II and ANX (n = 460) and those without ANX (n = 263)

Among individuals with BD-II (ANX *n* = 460; No ANX *n* = 263), similar differences emerged in pharmacotherapy patterns (**Supplementary Table 2**, Fig. 2). Individuals with BD-II + ANX were more likely to receive gabapentinoids (9.5% vs. 3.5%, *p* = 0.005) and benzodiazepines (40.3% vs. 24.1%, *p* < 0.001) compared to those without ANX. They were also significantly more likely to be prescribed SSRIs (31.8% vs. 18.9%, *p* < 0.001) and any antidepressant (62.6% vs. 43.9%, *p* < 0.001), including antidepressants without a mood stabilizer (19.2% vs. 13.0%, *p* = 0.046). Current use of lamotrigine (51.4% vs. 46.6%, *p* = 0.313) and valproic acid (21.0% vs. 24.9%, *p* = 0.452) did not differ significantly, nor did prescriptions for carbamazepine, quetiapine, aripiprazole, risperidone, FGAs, or thyroid hormones.

Lifetime treatment patterns reflected similar trends: individuals with BD-II + ANX show a trend toward higher gabapentinoids use (15.0% vs. 8.5%, *p* = 0.015), benzodiazepines (57.2% vs. 37.7%, *p* < 0.001), and SNRIs (48.2% vs. 35.2%, *p*= 0.002). There was a trend toward higher use of quetiapine (39.0% vs. 30.2%, *p* = 0.030) and overall SGAs (64.2% vs. 51.7%, *p* = 0.003). In contrast, lifetime lithium and valproate use showed no significant differences.

### Effect of Comorbid ANX on Pharmacological Treatment Response in BD

Individuals with BD + ANX demonstrated lower treatment responses to lithium (4.91 vs. 6.05, p < 0.001), MSACs (5.16 vs. 6.01, *p* = 0.005), and SGAs (4.67 vs. 5.73, *p* < 0.001) compared to those with BD + NoANX, as measured by Alda A scores (Table 3). This pattern of reduced treatment response was evident in both BD-I and BD-II subtypes. However, the differences between groups were less pronounced in BD-II than in BD-I, and the bipolar II comparisons were further limited by smaller sample sizes.

## DISCUSSION

This study represents one of the largest systematic examinations of the clinical, demographic, and pharmacotherapeutic differences between individuals with BD + ANX and BD + NoANX. Our findings reveal that anxiety comorbidity in BD is associated with a more complex clinical presentation, greater psychiatric and somatic burden, distinct prescribing patterns, and poorer treatment response to mood stabilizers. These observations have important implications for clinical management and highlight the need for tailored therapeutic approaches in this phenotypically distinct subgroup.

### Clinical and Demographic Characteristics

Individuals with BD + ANX demonstrated a significantly more severe clinical profile compared to those of BD + NoANX. The higher prevalence of rapid cycling, suicide attempts, and multiple SUDs in the BD + ANX group suggests a more treatment-refractory illness course. Consistent with previous research showing that ANX emerge earlier and are more common in women (Simon et al., 2004; Vazquez et al., 2014), our BD + ANX group demonstrated younger age and female predominance. This pattern supports the hypothesis that anxiety may be an early predictor of bipolar disorder onset (McElroy et al., 2001). Our findings align with a prior systematic review showing greater SUD prevalence and poorer treatment response in individuals with BD and concurrent ANX (Vazquez et al., 2014). The association with a history of suicide attempts is consistent with data from the Systematic Treatment Enhancement Program for Bipolar Disorder (Simon et al., 2004), reinforcing that the anxiety phenotype reflects a more severe clinical profile. The substantially elevated rates of PTSD, BED, and OCD in the BD + ANX cohort further underscore the complex psychiatric burden characterizing this population.

The familial aggregation patterns observed in our study are particularly noteworthy. Individuals with BD + ANX had significantly higher rates of family history across multiple psychiatric conditions, including BD, anxiety, depression, and alcohol use disorder. This suggests potential shared genetic vulnerabilities (Williams et al., 2024)or environmental factors that predispose to both mood and anxiety pathology, consistent with emerging evidence of common neurobiological substrates underlying these disorders (Lopes et al., 2020; Maki et al., 2025). Both disorders share a core fronto-limbic network, characterized by overlapping amygdala hyperactivation and impaired prefrontal cortex regulation (Bi et al., 2022).

The increased medical comorbidity burden, as reflected by higher MCIRS scores and elevated migraine prevalence in the BD + ANX group, aligns with the growing recognition that psychiatric comorbidity often parallels increased somatic illness (Romo-Nava et al., 2021). The substantially higher medication burden —both current and lifetime—in the anxiety comorbid group likely reflects both the greater clinical complexity and the challenges in achieving symptom control in this population (Feske et al., 2000).

### Pharmacotherapeutic Patterns and Implications

A key finding of this study is the distinct pharmacotherapeutic patterns observed between individuals with and without comorbid anxiety. Individuals with BD + ANX were significantly less likely to receive lithium and valproic acid, medications with robust evidence for mood stabilization and suicide prevention in BD. Conversely, they were more likely to be prescribed monoaminergic antidepressants, including SSRIs and SNRIs, as well as adjunctive agents such as benzodiazepines and gabapentinoids. Our findings are consistent with prior reports showing higher rates of antidepressant prescriptions among individuals with BD + ANX (Galimberti et al., 2020; Keck et al., 2006; Vazquez et al., 2014). Notably, a substantial proportion of individuals with BD + ANX received one or more antidepressants without concurrent mood stabilizer coverage—a prescribing pattern that may increase the risk of affective instability, rapid cycling, and mood switching, particularly in BD-I. It remains unclear whether unimodal antidepressant use or lower rates of lithium and anticonvulsant mood stabilizer use confer greater risk, or whether anxiety comorbidity itself or associated substance use comorbidity drives illness severity measures such as cycle acceleration and mood switching (Altshuler et al., 1995).

The lower utilization of lithium and valproate in the BD + ANX group may reflect multiple factors. Clinicians may avoid these agents due to concerns about tolerability, narrow therapeutic windows, or monitoring requirements in patients already managing complex medication regimens. The reduced valproate prescribing may also reflect growing safety concerns, particularly in women of childbearing potential due to teratogenic risks (Freeman, 2022) and, more recently, emerging recommendations in European guidelines to exercise caution even in men (Singh et al., 2025b). Lamotrigine emerged as a notable exception, with slightly higher utilization in the BD + ANX group, particularly among those with BD-II. This pattern may reflect lamotrigine’s favorable tolerability profile and its efficacy in preventing depressive episodes (Keck et al., 2006). The preference for antidepressants may stem from attempts to target depressive and anxious symptoms concurrently, despite limited evidence supporting antidepressant monotherapy in BD (Elmosalamy et al., 2025). The increased prescription of benzodiazepines and gabapentinoids likely represents symptomatic management of anxiety, though benzodiazepines carry risks of dependence and may not address underlying mood dysregulation. The relative safety of gabapentinoids, and their evidence base for use in GAD and SAD, without increasing the risk of affective switching, highlight their potential role in this specific phenotype (Frye and Singh, 2024; Rickels et al., 2005). The observation that individuals with BD + ANX received significantly more lifetime medications underscores the complexity of treating this difficult-to-treat population.

### Treatment Response and Clinical Outcomes

Perhaps most clinically significant is the substantially poorer treatment response to mood stabilizers among individuals with BD + ANX, as measured by the Alda-A score. Mean response scores for lithium, MSACs, and SGAs were all significantly lower in the anxiety comorbid group across both BD subtypes, with the most pronounced differences in BD-I. These findings suggest anxiety comorbidity may represent a negative prognostic indicator associated with attenuated response to standard mood stabilizers. This finding aligns with previous research indicating that anxiety is a predictor of poor outcomes in BD (Feske et al., 2000; Gaudiano and Miller, 2005; Simon et al., 2004). This treatment resistance may reflect distinct neurobiological underpinnings—such as hyperactive fear circuitry, dysregulated stress response systems, or altered serotonergic, GABAergic, and dopaminergic neurotransmission—not adequately addressed by conventional mood stabilizers (Freeman et al., 2002). Additionally, chronic anxiety symptoms may perpetuate mood instability (Coryell et al., 2012)through behavioral mechanisms including sleep disruption and substance use as self-medication.

The BD + ANX group showed diminished lithium response, consistent with earlier reports of reduced efficacy in comorbid anxiety (Feske et al., 2000; Young et al., 1993), despite lithium’s antisuicidal effects. A recent open-label trial suggests lithium may improve comorbid anxiety in bipolar disorder, with similar effects at low (< 0.5) and high (> 0.5) doses, and improvements correlated with depressive symptoms (Jones et al., 2022). We lack lithium data and adherence information in our cohort, which should be addressed in future studies. However, randomized controlled trial evidence for this population remains limited (Kauer-Sant’Anna et al., 2009; Yatham et al., 2018). It is unclear whether anxiety comorbidity alters the neurobiology of BD, reducing lithium responsiveness, or whether factors such as suboptimal dosing, adherence challenges, or early discontinuation contribute. The cross-sectional design of this study limits causal conclusions, highlighting the need for future research using longitudinal treatment data and objective adherence measures.

### Subtype-Specific Considerations

Subgroup analyses revealed shared and distinct patterns in BD-I and BD-II with comorbid anxiety. In both subtypes, anxiety was associated with increased antidepressant use, higher rates of benzodiazepine and gabapentinoid prescriptions, and reduced lithium utilization. However, several differences emerged. Among individuals with BD-I, anxiety comorbidity was associated with significantly lower olanzapine use and reduced thyroid hormone supplementation. In contrast, BD-II individuals with anxiety showed higher lifetime use of SGAs, particularly quetiapine, possibly reflecting its dual FDA indication for both BD depression and GAD.

GAD was more prevalent in BD-II than BD-I, consistent with the depressive predominance of BD-II and the frequent co-occurrence of anxiety with depressive states. The particularly high rate of antidepressant monotherapy in BD-II individuals with anxiety (19.2% without mood stabilizer coverage) raises concerns about potential mood destabilization, even though the risk of treatment-emergent mania may be lower in BD-II than BD-I.

### Clinical and Research Implications

The findings of this study have several important clinical implications. First, the presence of comorbid ANX should be recognized as a marker of illness severity and treatment complexity in BD. Clinicians should maintain heightened vigilance for suicide risk, substance use, and somatic comorbidities in this population, implementing comprehensive assessment and monitoring strategies. Second, the prevalent use of antidepressant monotherapy in the BD + ANX group—particularly without mood stabilizer coverage—warrants critical reconsideration. While anxiety symptoms are distressing and merit treatment, antidepressant monotherapy in BD carries risks of mood destabilization. Current clinical guidelines recommend mood stabilizer therapy as the foundation of BD treatment, with adjunctive agents added as needed for residual symptoms. Our data suggest this principle may be frequently overlooked in individuals with prominent anxiety, potentially contributing to the poorer outcomes observed in this group. Third, the reduced treatment response to standard mood stabilizers in BD + ANX highlights the need for alternative or augmentation strategies. Potential approaches might include psychotherapy (particularly cognitive-behavioral therapy with anxiety-specific modules), optimization of mood stabilizer dosing before adding additional agents, or investigation of novel pharmacological targets. Short term (8 weeks) data support quetiapine as a helpful option for BD + ANX (Hirschfeld et al., 2006; Sheehan et al., 2013).

### Limitations

Several limitations of this study merit consideration. First, the cross-sectional design precludes causal inferences about the relationships between anxiety comorbidity, prescribing patterns, and treatment outcomes. Longitudinal studies tracking symptom trajectories and medication changes over time would provide more definitive insights. Second, anxiety diagnoses were determined at enrollment and may not reflect the full longitudinal course of anxiety symptoms, which can fluctuate with mood state. Third, our sample was predominantly recruited from U.S. sites and largely composed of white participants, with limited representation from Mexico and Chile, which may limit generalizability. Finally, we did not have detailed data on antidepressant treatment duration, dosing, or specific indications, which would help clarify whether these agents were prescribed primarily for anxiety, depression, or both.

## CONCLUSIONS

This large-scale study highlights that 60% of individuals with bipolar disorder have comorbid anxiety, forming a clinically distinct subgroup with greater psychiatric and somatic complexity, unique pharmacotherapy patterns, and reduced response to standard mood stabilizers. Widespread antidepressant use—often without adequate mood stabilizer coverage—and high benzodiazepine use raise concerns about current prescribing practices. These findings emphasize the need for anxiety-informed treatment strategies, further research into neurobiological mechanisms of treatment resistance, and more intensive monitoring and tailored interventions for this high-risk population.

## Supplementary Material

This is a list of supplementary files associated with this preprint. Click to download.

• Supplement.docx

## Figures and Tables

**Table 1:** Comparisons of demographic and clinical characteristics between individuals with BD, with and without ANX, adjusted for age, sex, and recruitment site.

	Total (*N* = 2225)	No ANX (*N* = 859)	ANX (*N* = 1366)	*p*-value
**Site of recruitment, n**	2225	859	1336	
Mayo, *n* (%)	1154 (51.9%)	495 (57.6%)	659 (48.2%)	< 0.001*
Lindner, *n* (%)	770 (34.6%)	219 (25.5%)	551 (40.3%)	
University of Minnesota, *n* (%)	75 (3.4%)	29 (3.4%)	46 (3.4%)	
Chile, *n* (%)	75 (3.4%)	51 (5.9%)	24 (1.8%)	
Mexico, *n* (%)	151 (6.8%)	65 (7.6%)	86 (6.3%)	
**Age, n**	2219	856	1363	
Mean (SD)	41.6 (15.0)	43.6 (15.9)	40.4 (14.3)	**< 0.001** *
**Sex, n**	2225	859	1366	
Male, *n* (%)	844 (37.9%)	388 (45.2%)	456 (33.4%)	**< 0.001** *
Female, *n* (%)	1381 (62.1%)	471 (54.8%)	910 (66.6%)	
**Race, n**	2191	840	1351	
White, *n* (%)	1840 (84.0%)	705 (83.9%)	1135 (84.0%)	0.005*
Black, *n* (%)	62 (2.8%)	18 (2.1%)	44 (3.3%)	
Asian, *n* (%)	22 (1.0%)	15 (1.8%)	7 (0.5%)	
Other, *n* (%)	170 (7.8%)	73 (8.7%)	97 (7.2%)	
Multiracial, *n* (%)	97 (4.4%)	29 (3.5%)	68 (5.0%)	
**Hispanic, n**	2179	833	1346	
Yes, *n* (%)	271 (12.4%)	124 (14.9%)	147 (10.9%)	0.223
**Body mass index, n**	2118	818	1300	
Mean (SD)	29.9 (7.4)	29.4 (6.8)	30.2 (7.7)	0.094
**Currently married, n**	2201	848	1353	
Yes, *n* (%)	997 (45.3%)	403 (47.5%)	594 (43.9%)	0.821
**Employment, n**	2106	809	1297	
Full-time, *n* (%)	544 (25.8%)	226 (27.9%)	318 (24.5%)	0.012
Part-time, *n* (%)	401 (19.0%)	170 (21.0%)	231 (17.8%)	
Not working for pay at present, *n* (%)	1161 (55.1%)	413 (51.1%)	748 (57.7%)	
**Education, n**	2134	814	1320	
High school or less, *n* (%)	61 (2.9%)	26 (3.2%)	35 (2.7%)	0.271
High school graduated, *n* (%)	284 (13.3%)	97 (11.9%)	187 (14.2%)	
Beyond high school graduation, *n* (%)	1789 (83.8%)	691 (84.9%)	1098 (83.2%)	
Bipolar Disorder Characteristics				
**Bipolar type, n**	2225	859	1366	
Bipolar I, *n* (%)	1451 (65.2%)	573 (66.7%)	878 (64.3%)	0.234
Bipolar II, *n* (%)	723 (32.5%)	263 (30.6%)	460 (33.7%)	
Schizoaffective, *n* (%)	51 (2.3%)	23 (2.7%)	28 (2.0%)	
**Rapid cycling, n**	2083	783	1300	
Yes, *n* (%)	1189 (57.1%)	354 (45.2%)	835 (64.2%)	**< 0.001**
**History of psychosis, n**	2207	852	1355	
Yes, *n* (%)	874 (39.6%)	352 (41.3%)	522 (38.5%)	0.302
**Manic psychosis, n**	2207	852	1355	
Yes, *n* (%)	693 (31.4%)	301 (35.3%)	392 (28.9%)	0.012
**Early onset (≤ 19 years old), n**	2094	791	1303	
Yes, *n* (%)	797 (38.1%)	275 (34.8%)	522 (40.1%)	0.016
**Suicide attempt, n**	2211	854	1357	
Yes, *n* (%)	760 (34.4%)	212 (24.8%)	548 (40.4%)	**< 0.001**
**Suicide attempt (≤ 19 years old), n**	571	156	415	
Yes, *n* (%)	256 (44.8%)	66 (42.3%)	190 (45.8%)	0.457
Lifetime Psychiatric Illness History				
**Child ADHD, n**	2185	853	1332	
Yes, *n* (%)	347 (15.9%)	110 (12.9%)	237 (17.8%)	0.015
**Anorexia or bulimia, n**	2200	857	1343	
Yes, *n* (%)	194 (8.8%)	50 (5.8%)	144 (10.7%)	0.005
**Binge eating disorder, n**	2202	855	1347	
Yes, *n* (%)	270 (12.3%)	64 (7.5%)	206 (15.3%)	**< 0.001**
**Post-traumatic stress disorder, n**	2202	855	1347	
Yes, *n* (%)	574 (26.1%)	108 (12.6%)	466 (34.6%)	**< 0.001**
**Obsessive compulsive disorder, n**	2196	857	1339	
Yes, *n* (%)	358 (16.3%)	73 (8.5%)	285 (21.3%)	**< 0.001**
Lifetime Substance Use Disorder				
**Substance use disorder, n**	2135	836	1299	
Yes, *n* (%)	1283 (60.1%)	458 (54.8%)	825 (63.5%)	**< 0.001**
**Tobacco use disorder, n**	2200	854	1346	
Yes, *n* (%)	880 (40.0%)	300 (35.1%)	580 (43.1%)	**< 0.001**
**Alcohol use disorder, n**	2207	856	1351	
Yes, *n* (%)	879 (39.8%)	308 (36.0%)	571 (42.3%)	0.002
**Cocaine use disorder, n**	2193	852	1341	
Yes, *n* (%)	313 (14.3%)	90 (10.6%)	223 (16.6%)	**< 0.001**
**Cannabis use disorder, n**	2205	855	1350	
Yes, *n* (%)	658 (29.8%)	209 (24.4%)	449 (33.3%)	**< 0.001**
**Methamphetamine use disorder, n**	2200	855	1345	
Yes, *n* (%)	200 (9.1%)	60 (7.0%)	140 (10.4%)	0.002
**Opioid use disorder, n**	2189	854	1335	
Yes, *n* (%)	230 (10.5%)	59 (6.9%)	171 (12.8%)	**< 0.001**
Family History (First-degree Relatives)				
**Bipolar disorder, n**	1692	648	1044	
Yes, *n* (%)	799 (47.2%)	261 (40.3%)	538 (51.5%)	**< 0.001**
**Anxiety, n**	722	296	426	
Yes, *n* (%)	439 (60.8%)	147 (49.7%)	292 (68.5%)	**< 0.001**
**Depression, n**	1877	707	1170	
Yes, *n* (%)	1505 (80.2%)	513 (72.6%)	992 (84.8%)	**< 0.001**
**Alcohol use disorder, n**	1894	721	1173	
Yes, *n* (%)	928 (49.0%)	294 (40.8%)	634 (54.0%)	**< 0.001**
**Suicide, n**	1873	721	1152	
Yes, *n* (%)	167 (8.9%)	55 (7.6%)	112 (9.7%)	0.124
**MCIRS, mean (SD)**	6.191 (6.570)	5.421 (6.207)	6.676 (6.746)	**< 0.001**
**Medical comorbidities, n**	758	312	446	
Hypertension, *n* (%)	130 (17.2%)	52 (16.7%)	78 (17.5%)	0.522
Musculoskeletal, integumentary, *n* (%)	6 (0.8%)	2 (0.6%)	4 (0.9%)	0.707
Eczema, *n* (%)	69 (9.1%)	27 (8.7%)	42 (9.4%)	0.275
Psoriasis, *n* (%)	24 (3.2%)	10 (3.2%)	14 (3.1%)	0.721
Vitiligo, *n* (%)	4 (0.5%)	2 (0.6%)	2 (0.4%)	0.390
Diabetes, *n* (%)	58 (7.7%)	21 (6.7%)	37 (8.3%)	0.193
PCOS, *n* (%)	40 (5.3%)	11 (3.5%)	29 (6.5%)	0.172
Thyroid, *n* (%)	135 (17.8%)	61 (19.6%)	74 (16.6%)	0.734
Rheum arthritis, *n* (%)	14 (1.8%)	5 (1.6%)	9 (2.0%)	0.529
Fibromyalgia, *n* (%)	44 (5.8%)	11 (3.5%)	33 (7.4%)	0.068
Stroke, *n* (%)	4 (0.5%)	1 (0.3%)	3 (0.7%)	0.882
Epilepsy, *n* (%)	50 (6.6%)	11 (3.5%)	39 (8.7%)	0.038
Obstructive sleep apnea, *n* (%)	82 (10.8%)	32 (10.3%)	50 (11.2%)	0.666
Migraines, *n* (%)	223 (29.4%)	59 (18.9%)	164 (36.8%)	**< 0.001**
Osteoporosis, *n (*%)	31 (4.1%)	7 (2.2%)	24 (5.4%)	0.010
Osteoarthritis, *n* (%)	94 (12.4%)	28 (9.0%)	66 (14.8%)	0.098
COPD, *n* (%)	23 (3.0%)	8 (2.6%)	15 (3.4%)	0.345
**Obese, n**	2118	818	1300	
Yes, *n* (%)	888 (41.9%)	317 (38.8%)	571 (43.9%)	0.140
**Current psychotropics, n**	2216	854	1362	
Mean (SD)	2.723 (1.581)	2.502 (1.469)	2.861 (1.632)	**< 0.001**
**Lifetime psychotropics, n**	2216	854	1362	
Mean (SD)	6.681 (5.456)	5.737 (4.765)	7.273 (5.772)	**< 0.001**
**Antidepressant-induced mania, n**	1848	672	1176	0.207
Mean (SD)	384 (20.8%)	118 (17.6%)	266 (22.6%)	
**Tardive dyskinesia, n**	729	293	436	
Yes, *n* (%)	67 (9.2%)	25 (8.5%)	42 (9.6%)	0.391

* Not adjusted for age, sex, and site of recruitment.

**Table 2:** Differences in pharmacotherapeutic prescriptions among individuals with bipolar disorder, with and without comorbid anxiety (ANX).

	Total (*N* = 2225)	No ANX (*N* = 859)	ANX (*N* = 1366)	*p*-value*
***Current Prescriptions***				
**Lithium, n**	1516	579	937	0.005
Yes, *n* (%)	625 (41.2%)	277 (47.8%)	348 (37.1%)	
**Lamotrigine, n**	1700	628	1072	0.027
Yes, *n* (%)	651 (38.3%)	225 (35.8%)	426 (39.7%)	
**Valproic acid, n**	1638	631	1007	0.047
Yes, *n* (%)	406 (24.8%)	187 (29.6%)	219 (21.7%)	
**Carbamazepine, n**	1543	578	965	0.807
Yes, *n* (%)	43 (2.8%)	15 (2.6%)	28 (2.9%)	
**Gabapentinoids, n**	2198	848	1350	**< 0.001**
Yes, *n* (%)	153 (7.0%)	38 (4.5%)	115 (8.5%)	
**Benzodiazepines, n**	1985	743	1242	**< 0.001**
Yes, *n* (%)	693 (34.9%)	198 (26.6%)	495 (39.9%)	
**Non-BZD sedatives, n**	1974	740	1234	0.387
Yes, *n* (%)	154 (7.8%)	52 (7.0%)	102 (8.3%)	
**SGA, n**	2092	801	1291	0.635
Yes, *n* (%)	1110 (53.1%)	418 (52.2%)	692 (53.6%)	
**Olanzapine, n**	1544	601	943	0.008
Yes, *n* (%)	159 (10.3%)	84 (14.0%)	75 (8.0%)	
**Clozapine, n**	1514	585	929	0.740
Yes, *n* (%)	23 (1.5%)	9 (1.5%)	14 (1.5%)	
**Quetiapine, n**	1625	620	1005	0.141
Yes, *n* (%)	461 (28.4%)	171 (27.6%)	290 (28.9%)	
**Aripiprazole, n**	1566	599	967	0.353
***Current Prescriptions***				
Yes, *n* (%)	250 (16.0%)	84 (14.0%)	166 (17.2%)	
**Risperidone, n**	1551	597	954	0.735
Yes, *n* (%)	128 (8.3%)	48 (8.0%)	80 (8.4%)	
**FGA, n**	1583	602	981	0.388
Yes, *n* (%)	25 (1.6%)	12 (2.0%)	13 (1.3%)	
**Any antidepressants, n**	2102	783	1319	**< 0.001**
Yes, *n* (%)	1018 (48.4%)	309 (39.5%)	709 (53.8%)	
**Two or more antidepressants, n**	2102	783	1319	**<0.001**
Yes, *n* (%)	226 (10.8%)	54 (6.9%)	172 (13.0%)	
**SSRI, n**	2078	772	1306	**< 0.001**
Yes, *n* (%)	506 (24.4%)	130 (16.8%)	376 (28.8%)	
**SNRI, n**	1764	656	1108	0.254
Yes, *n* (%)	228 (12.9%)	77 (11.7%)	151 (13.6%)	
**TCA, n**	1894	684	1210	0.140
Yes, *n* (%)	83 (4.4%)	26 (3.8%)	57 (4.7%)	
**Antidepressant without MS, n**	2160	828	1332	**< 0.001**
Yes, *n* (%)	311 (14.4%)	80 (9.7%)	231 (17.3%)	
**Thyroid hormone, n**	1185	431	754	0.067
Yes, *n* (%)	262 (22.1%)	113 (26.2%)	149 (19.8%)	
**Stimulants/wakefulness agents, n**	1675	610	1065	0.939
Yes, *n* (%)	199 (11.9%)	67 (11.0%)	132 (12.4%)	
**Dopamine agonist, n**	1684	618	1066	0.769
Yes, *n* (%)	24 (1.4%)	9 (1.5%)	15 (1.4%)	
**Two or more SGA, n**	2092	801	1291	0.430
Yes, *n* (%)	91 (4.3%)	39 (4.9%)	52 (4.0%)	
***Current Prescriptions***				
**One or more MS, n**	2165	833	1332	0.060
Yes, *n* (%)	1503 (69.4%)	617 (74.1%)	886 (66.5%)	
**Two or more MSs, n**	2165	833	1332	0.597
Yes, *n* (%)	220 (10.2%)	87 (10.4%)	133 (10.0%)	
**Three or more MSs, n**	2165	833	1332	0.957
Yes, *n* (%)	2 (0.1%)	0 (0.0%)	2 (0.2%)	
**No MS, n**	2165	833	1332	0.060
Yes, *n* (%)	662 (30.6%)	216 (25.9%)	446 (33.5%)	
**No medications, n**	2216	854	1362	0.762
Yes, *n* (%)	183 (8.3%)	62 (7.3%)	121 (8.9%)	
**Lifetime Prescriptions**				
**Lithium ever, n**	1876	717	1159	0.030
Yes, *n* (%)	1009 (53.8%)	415 (57.9%)	594 (51.3%)	
**Lamotrigine ever, n**	2063	781	1282	0.150
Yes, *n* (%)	880 (42.7%)	310 (39.7%)	570 (44.5%)	
**Valproate ever, n**	2071	783	1288	0.013
Yes, *n* (%)	769 (37.1%)	323 (41.3%)	446 (34.6%)	
**Gabapentinoids ever, n**	2198	848	1350	**< 0.001**
Yes, *n* (%)	286 (13.0%)	75 (8.8%)	211 (15.6%)	
**Benzodiazepines ever, n**	1985	743	1242	**< 0.001**
Yes, *n* (%)	1060 (53.4%)	330 (44.4%)	730 (58.8%)	
**FGA ever, n**	1583	602	981	0.897
n (%)	163 (10.3%)	66 (11.0%)	97 (9.9%)	
**SGA ever, n**	2092	801	1291	0.172
***Current Prescriptions***				
n (%)	1457 (69.6%)	534 (66.7%)	923 (71.5%)	
**Olanzapine ever, n**	2043	788	1255	0.034
Yes, *n* (%)	374 (18.3%)	171 (21.7%)	203 (16.2%)	
**Quetiapine ever, n**	2050	788	1262	0.010
Yes, *n* (%)	787 (38.4%)	274 (34.8%)	513 (40.6%)	
**Aripiprazole ever, n**	2042	785	1257	0.386
n (%)	543 (26.6%)	178 (22.7%)	365 (29.0%)	
**Risperidone ever, n**	2045	789	1256	0.773
n (%)	419 (20.5%)	153 (19.4%)	266 (21.2%)	
**Any antidepressant ever, n**	2102	783	1319	**< 0.001**
n (%)	1710 (81.4%)	590 (75.4%)	1120 (84.9%)	
**SSRI ever, n**	2078	772	1306	**< 0.001**
Yes, *n* (%)	1373 (66.1%)	452 (58.5%)	921 (70.6%)	
**SNRI ever, n**	1764	656	1108	0.002
Yes, *n* (%)	644 (36.5%)	200 (30.5%)	444 (40.1%)	
**TCA ever, n**	1894	684	1210	0.012
Yes, *n* (%)	350 (18.5%)	105 (15.4%)	245 (20.2%)	
**Antidepressant without MS ever, n**	2164	831	1333	**< 0.001**
n (%)	267 (12.3%)	72 (8.7%)	195 (14.6%)	
**Thyroid hormone ever, n**	1185	431	754	0.300
n (%)	120 (10.1%)	51 (11.8%)	69 (9.2%)	
**Stimulants/wakefulness agents ever, n**	1675	610	1065	0.060
n (%)	432 (25.8%)	129 (21.1%)	303 (28.5%)	
**Dopamine agonist ever, n**	1684	618	1066	0.736
***Current Prescriptions***				
n (%)	44 (2.6%)	17 (2.8%)	27 (2.5%)	
**Two or more SGAs ever, n**	2092	801	1291	0.866
n (%)	640 (30.6%)	229 (28.6%)	411 (31.8%)	
**One or more MS ever, n**	2165	833	1332	0.043
n (%)	1799 (83.1%)	714 (85.7%)	1085 (81.5%)	
**Two or more MSs ever, n**	2165	833	1332	0.873
n (%)	721 (33.3%)	279 (33.5%)	442 (33.2%)	
**Three or more MSs ever, n**	2165	833	1332	0.415
n (%)	249 (11.5%)	101 (12.1%)	148 (11.1%)	
**No MS ever, n**	2165	833	1332	0.043
n (%)	366 (16.9%)	119 (14.3%)	247 (18.5%)	
**No medications ever, n**	2216	854	1362	0.827
n (%)	36 (1.6%)	15 (1.8%)	21 (1.5%)	

* Models adjusted for age, sex, and recruitment site.

ANX: anxiety disorders; BZD: benzodiazepines; FGA: first-generation antipsychotics; MS: mood stabilizer (includes valproate, lamotrigine, carbamazepine, and lithium); SGA: second-generation antipsychotic; SNRI: serotonin-norepinephrine reuptake inhibitor; SSRI: selective serotonin reuptake inhibitor; TCA: tricyclic antidepressant.

**Table 3:** Treatment responses of lithium, mood-stabilizing anticonvulsants (MSACs) and second-generation antipsychotics (SGAs), measured by Alda A scores, in individuals with bipolar disorder with and without comorbid anxiety (ANX).

	All*			BD-I			BD-II		
	No ANX	ANX	*p*-value	No ANX	ANX	*p*-value	No ANX	ANX	*p*-value
**Lithium response (Alda-A), n**	289	404	**< 0.001**	218	300	0.004	66	92	0.183
Mean (SD)	6.05 (2.65)	4.91 (2.93)		6.28 (2.59)	5.07 (2.88)		5.21 (2.78)	4.39 (3.03)	
**MSACs (Alda-A), n**	232	387	0.005	153	248	0.011	78	138	0.165
Mean (SD)	6.01 (2.56)	5.16 (2.59)		6.12 (2.46)	5.08 (2.53)		5.77 (2.76)	5.26 (2.67)	
**SGAs (Alda-A), n**	230	309	**< 0.001**	131	257	0.002	46	100	0.080
Mean (SD)	5.73 (2.56)	4.67 (2.69)		5.87 (2.58)	4.69 (2.70)		5.48 (2.51)	4.65 (2.71)	

* Including BD-I, BD-II and schizoaffective (bipolar type).

## Data Availability

The datasets used and/or analyzed during the current study are available from the corresponding author on reasonable request.
